# mSphere of Influence: Deciphering purine auxotrophy in protozoan parasites

**DOI:** 10.1128/msphere.00007-24

**Published:** 2024-04-03

**Authors:** Bruno Martorelli Di Genova

**Affiliations:** 1 Department of Microbiology and Molecular Genetics, Larner College of Medicine, University of Vermont, Burlington, Vermont, USA; University at Buffalo, Buffalo, New York, USA

**Keywords:** apicomplexan parasites, purine metabolism, protozoa, *Leishmania*, *Plasmodium falciparum*

## Abstract

Bruno Martorelli Di Genova works in parasitology, focusing on *Toxoplasma gondii* metabolism. In this mSphere of Influence article, he reflects on how the articles “Metabolic Reprogramming during Purine Stress in the Protozoan Pathogen *Leishmania donovani*” and “Yeast-Based High-Throughput Screen Identifies *Plasmodium falciparum* Equilibrative Nucleoside Transporter 1 Inhibitors That Kill Malaria Parasites” impacted him, informing his research strategies and understanding of metabolic flexibility in *Toxoplasma gondii*.

## COMMENTARY

*De novo* nucleic acid synthesis and salvage pathways are crucial for cellular growth and survival. In higher eukaryotes, various cellular pathways regulate nucleoside transport, salvage, and metabolism by integrating intracellular and extracellular signals (1, 2). In contrast, protozoan parasites have undergone evolutionary adaptations, losing the ability to synthesize purines. Purine auxotrophy is observed across all protozoan parasite lineages, requiring these parasites to acquire purines from their host to ensure survival (3). A seminal study that sheds light on this phenomenon is “Metabolic Reprogramming during Purine Stress in the Protozoan Pathogen *Leishmania donovani*” by Martin et al. (4). This research meticulously examines how *Leishmania donovani*, the causative agent of leishmaniasis, alters its protein expression in response to purine starvation, mimicking a nutrient-scarce environment within a host. The study employs an accurate mass and time (AMT) tag approach for proteomics, enabling the precise quantification of proteome dynamics. The findings reveal a specific upregulation of enzymes and transporters involved in purine salvage pathways, highlighting the organism’s strategy for adapting to nutrient stress at the molecular level. This study exemplifies the power of advanced proteomics in uncovering the complexities of metabolic adaptation and underscores the central role of purine auxotrophy in host-parasite interactions.

Building on the concept of purine auxotrophy, another study, titled “Yeast-Based High-Throughput Screen Identifies Plasmodium falciparum Equilibrative Nucleoside Transporter 1 Inhibitors That Kill Malaria Parasites,” explores the potential of nucleoside transporters as a drug *Plasmodium falciparum* target (5). Frame et al. employed a yeast-based high-throughput screen to identify inhibitors of the *P. falciparum* Equilibrative Nucleoside Transporter 1 (PfENT1), a previously identified nucleoside transporter. By exploiting the sensitivity of *Saccharomyces cerevisiae* to the cytotoxic nucleoside analog 5-fluorouridine (5-FUrd), they develop a robust yeast growth assay to detect PfENT1 inhibitors. This innovative approach identified 171 compounds, further validated through secondary assays involving yeast and *P. falciparum* cultures.

In conclusion, these studies collectively illuminate the multifaceted implications of purine auxotrophy in protozoan parasites, revealing how this metabolic constraint not only influences the intricate dynamics of host-parasite interactions but also presents promising opportunities for the development of novel therapeutic strategies targeting this vulnerability.
